# Protein Tyrosine Phosphatase 4A3 (PTP4A3) Is Required for *Xenopus laevis* Cranial Neural Crest Migration *In Vivo*

**DOI:** 10.1371/journal.pone.0084717

**Published:** 2013-12-23

**Authors:** Selma Maacha, Nathalie Planque, Cécile Laurent, Caterina Pegoraro, Océane Anezo, Frédérique Maczkowiak, Anne H. Monsoro-Burq, Simon Saule

**Affiliations:** 1 Institut Curie, Research Division, Orsay, France; 2 CNRS UMR3347, Orsay, France; 3 INSERM U1021, Orsay, France; 4 Université Paris Sud, Orsay, France; 5 Université Paris Diderot, Sorbonne Paris Cité, France; University of Colorado, Boulder, United States of America

## Abstract

Uveal melanoma is the most common intraocular malignancy in adults, representing between about 4% and 5% of all melanomas. High expression levels of Protein Tyrosine Phosphatase 4A3, a dual phosphatase, is highly predictive of metastasis development and PTP4A3 overexpression in uveal melanoma cells increases their *in vitro* migration and *in vivo* invasiveness. Melanocytes, including uveal melanocytes, are derived from the neural crest during embryonic development. We therefore suggested that PTP4A3 function in uveal melanoma metastasis may be related to an embryonic role during neural crest cell migration. We show that PTP4A3 plays a role in cephalic neural crest development in *Xenopus laevis*. PTP4A3 loss of function resulted in a reduction of neural crest territory, whilst gain of function experiments increased neural crest territory. Isochronic graft experiments demonstrated that PTP4A3-depleted neural crest explants are unable to migrate in host embryos. Pharmacological inhibition of PTP4A3 on dissected neural crest cells significantly reduced their migration velocity *in vitro*. Our results demonstrate that PTP4A3 is required for cephalic neural crest migration *in vivo* during embryonic development.

## Introduction

Uveal melanoma (UM), which results from a malignant transformation of uveal melanocytes (located in the iris, ciliary body and choroid), is the most common intraocular malignancy in adults. It represents about 4% or 5% of all melanomas. Although the disease is limited to the eye in over 95% of patients at diagnosis, about 50% will develop metastases after a median time of three years [1,2]. Gene expression profiling (GEP) or transcriptomic analysis studies have identified two major subgroups of UM, one of low and one of high metastatic potential [3]. Using these transcriptomic approaches, we previously showed that high expression of PTP4A3 also called PRL-3 (Protein Tyrosine Phosphatase 4A3/ Protein of Regenerating Liver-3), a dual phosphatase encoding gene [4], is predictive of metastasis development [5]. PTP4A3 overexpression in UM cells increased their *in vitro* migration and *in vivo* invasiveness [1]. 

 PTP4A3 forms the family of PTP4A phosphatases with PTP4A1 and PTP4A2. These PTPases are closely related since they share 75% amino acid sequence identity. Their sequence contains a PTP active site signature (CX_5_R) and a C-terminal CAAX prenylation sequence. Considerable evidence suggests a causal role for PTP4A3 in tumor metastasis [6] but little is known about PTP4A3-mediated cellular signaling pathways. Understanding of the physiological role of PTP4A3 during development is also limited. Expression of PTP4A genes during embryogenesis has been reported in *Drosophila* sp., *Amphioxus sp*. and zebrafish [7]. PTP4A3 is proposed to be more specific to the mesodermal lineage [7]. We previously demonstrated that PTP4A3 overexpression in transformed uveal melanocytes increased their migration and invasiveness and therefore chose to investigate if PTP4A3 could also have a role in the embryonic development of these cells. 

 Melanocytes derive from the neural crest (NC), a transient embryonic cell population that migrates extensively and differentiates into derivatives including melanocytes, most of the craniofacial skeleton and peripheral nervous system [8]. Uveal melanocytes are specifically produced from the cephalic NC [9]. NC cell (NCC) migration is accompanied by cell–cell adhesion modifications, cytoskeletal rearrangements and morphological changes that enable them to emigrate from the neural tube. The NCCs acquire cell-surface receptors, metalloproteases (MMP) and adhesion molecules that make them able to respond to cell–cell interactions and environmental cues that influence their migration pathways [8]. PTP4A3 is implicated in cell adhesion and the regulation of focal adhesion components including integrin beta-1, Src, paxillin and p130Cas [10,11]. Similarly, PTP4A3 promotes cell invasion by increasing MMP2 activity and decreasing the expression of the MMP inhibitor, TIMP2 [11]. PTP4A3 is also involved in the epithelial-mesenchymal transition (EMT) as its overexpression in colorectal carcinoma cells, leads to the downregulation of epithelial markers (E-cadherin, plakoglobin, and integrin beta-3) whilst upregulating expression of mesenchymal markers (fibronectin and snail1) [12]. 

 Some phosphatases are involved both in cancer progression and in NC development. For example, knockdown of the protein tyrosine phosphatase SHP2, encoded by PTPN11 in established breast tumors, blocked their growth and reduced metastasis [13]. In parallel, SHP2 is expressed in NCCs and is essential for their normal migration and differentiation into diverse lineages such as cardiac and craniofacial NC [14]. 

 To understand the role of PTP4A3 during NC development, we used *Xenopus laevis* as a model. *Xenopus laevis* model is widely used because of its tractability (large, abundant eggs and easily manipulated embryos) and close evolutionary relationship with humans as well as conserved cellular, developmental and genomic organization with mammals. Also, *Xenopus laevis* is the only vertebrate model system that allows high-throughput *in vivo* analysis of gene function providing insights into a multitude of human diseases and their potential treatments [15]. After establishing PTP4A3 expression pattern during early development in *Xenopus laevis* embryos, we conducted gain and loss of function experiments *in vivo*, by microinjecting either PTP4A3 mRNA or morpholinos to target PTP4A3 protein translation. Early *Xenopus laevis* embryos were injected and these were then allowed to grow until key stages of NC development. We assessed NC cell migration after isochronic grafting experiments *in vivo*. We also assessed NC migration velocity *in vitro* using pharmacological PTP4A3 inhibition. Our study demonstrates that PTP4A3 is expressed in the NC and that its function is required for the NC migration *in vivo*. 

## Materials and Methods

### Embryos and explants

*Xenopus laevis* embryos were obtained by *in vitro* fertilization and were grown, collected and fixed according to standard procedures [16]. Cranial NC explants were dissected from embryos at stage 17 (staging according to Nieuwkoop and Faber developmental table, 1994) [17]. 

### Microinjection, gain and loss of function experiments

Full length *Xenopus laevis PTP4A3* cDNA (Accession #NM_001091784.1) was isolated and tagged with an HA motif by PCR using a forward primer (5’ GCCACCATGTACCCATACGATGTTCCAGATTACGCTTACCCATACGATGTTCCAGATTAC GCTGCTCGTATCAATCGTCC 3’) and a reverse primer (5’ CGGCGGTCACATGATGCAGCATTTGTTCTTGTGGTTGTGAGGGTCCTTGAATCGG 3’). The PCR fragment obtained was then cloned using the TOPO TA Cloning kit (Invitrogen). The pCRII-TOPO-xlPTP4A3 template was used to synthesize xlPTP4A3 capped mRNA using the mMESSAGE mMachine kit (Ambion). The synthesized mRNA was then polyadenylated *in vitro* using the poly(A) tailing kit (Ambion). *Xenopus laevis* embryos at the two-cell or four-cell stage were injected into one dorsal animal blastomere with xlPTP4A3 mRNA (1ng), mouse mmPTP4A3 (1ng) or an inactive mutant form, mmPTP4A3(C104S) (1ng). For loss of function experiments, the antisense morpholino oligonucleotide (Gene Tools, Philomath, OR) directed against *X. laevis* PTP4A3 (referred to as xlPTP4A3-MO, 40ng) was CAGGACGATTGATACGAGCCATGAT and its mismatching control (referred to as 5MM-MO, 40ng) was CAGcACcATTcATACcAcCCATGAT. The morpholino-induced phenotype was rescued by the co-injection of mmPTP4A3 mRNA (1ng). The antisense morpholino oligonucleotide efficiency was validated using *in vitro* TNT**^*®*^** coupled reticulocyte lysate system (Promega) in the presence of radiolabeled S35 methionine (Perkinelmer) followed by SDS-PAGE. The lineage of the injected cell was monitored in each case by co-injection of either nuclear-targeted β-galactosidase mRNA (250 pg) or histone 2b-GFP mRNA (125 pg) [18,19]. 

Quantification of the loss of function phenotype was determined by measuring the height of the NC migratory streams using ImageJ software, while gain of function phenotype was quantified by measuring the area of the NC migratory streams using the same ImageJ software. 

### Isotopic and isochronic graft experiments

Stage 17 cranial NC explants, taken from embryos previously injected with either nuclear histone2b-EGFP mRNA (250pg) [19] and xlPTP4A3-MO or histone2b-EGFP mRNA only, were dissected. The NC explant was orthotopically grafted into a stage-matched sibling host embryo after unilateral ablation of the host cephalic NC. Grafted NC migration was analyzed from stage 22 to stage 25. Pictures were taken using a Scion camera on a Leica MZFLIII stereoscope and imported using PhotoshopCS software.

### Whole mount *in situ* hybridization and sectioning

The 5’-UTR region of xlPTP4A3 mRNA (Accession #NM_001091784.1) was isolated by PCR using a forward primer (5’ GAGTAGATTGGATTGATTCAGCAGTCGC 3’) and a reverse primer (5’ GTCCTCCTTCCTTACATCAGGAC 3’), and then cloned using the TOPO TA Cloning kit (Invitrogen). The pCRII-TOPO-5’UTR-xlPTP4A3 template was used to synthesize digoxigenin-labeled cRNA and generate antisense RNA probes *in vitro*. Embryos and explants were processed following whole-mount in situ hybridization (WISH) [17,20]. Sections (50–60 μm thick) were cut using a Leica VT1000 vibratome after gelatin-albumin embedding. Pictures were captured using a Scion camera on a Leica MZFLIII stereoscope and Olympus microscope and imported using ImageJ or Photoshop CS software. Scan levels were homogenized using level and brightness options in PhotoshopCS. Snai2 and Twist antisense probes were synthesized as described above [21,22].

### Cell proliferation and apoptosis immunodetection

Cell proliferation or apoptosis were assessed by immunodetection of phosphoHistone 3 (Ser10) and cleaved caspase 3, respectively. The bleached embryos were saturated in 10% goat serum then incubated in 1:500 polyclonal phosphoHistone H3 (Ser10) (Millipore, #06-570) or polyclonal cleaved caspase 3 (Cell Signalling, #9661) before washes, incubation in 1:500 phosphatase alkaline-conjugated secondary antibody and NBT-BCIP staining.

### Time-lapse videomicroscopy

Stage 17 cranial NC explants were dissected and cultured on fibronectin in a modified Danilchick medium containing 53mM NaCl, 5mM Sodium carbonate, 4.5mM Potassium gluconate, 32mM Sodium gluconate, 1mM Magnesium sulfate, 1mM Calcium chloride, 0.1% BSA, 5mM Bicine (pH 8.3) and 50 µg/ml gentamicine sulfate [23]. NCCs were treated with a specific inhibitor of PTP4A3 (PRL-3 Inhibitor I, Sigma) at a final concentration of 2 µM and the control NCCs were treated with vehicle (DMSO). Cranial NCC migration was monitored by time-lapse videomicroscopy at room temperature under bright light. This required an inverted phase contrast microscope (Leica DM IRB) with a charge coupled device (CCD) CoolSNAP camera (Roper Scientific Photometrics). Images were taken every 4min for 10h using Metamorph software. Movies were reconstructed with a plug-in for the ImageJ software (http://rsbweb.nih.gov/ij/) developed by F. Cordelières at Institut Curie (Orsay, France). Cells were tracked manually and the parameters calculated using another plug-in developed by F. Cordelières. 

### Statistical analyses

Data are expressed as mean ± SEM and analyzed by Microsoft Excel or StatView software. Differences between embryos phenotype in the loss of function experiment were examined with the Chi-squared test using Microsoft Excel whereas differences in the migratory streams length or area between the different groups of embryos were analyzed with the Mann Whitney test using StatView software.

Similarly, differences in migration velocity or pausing average between the different cells used were examined with the Mann Whitney test using StatView software.

## Results

### Spatiotemporal expression pattern of PTP4A3 in *X. laevis* embryos

**Figure 1:**
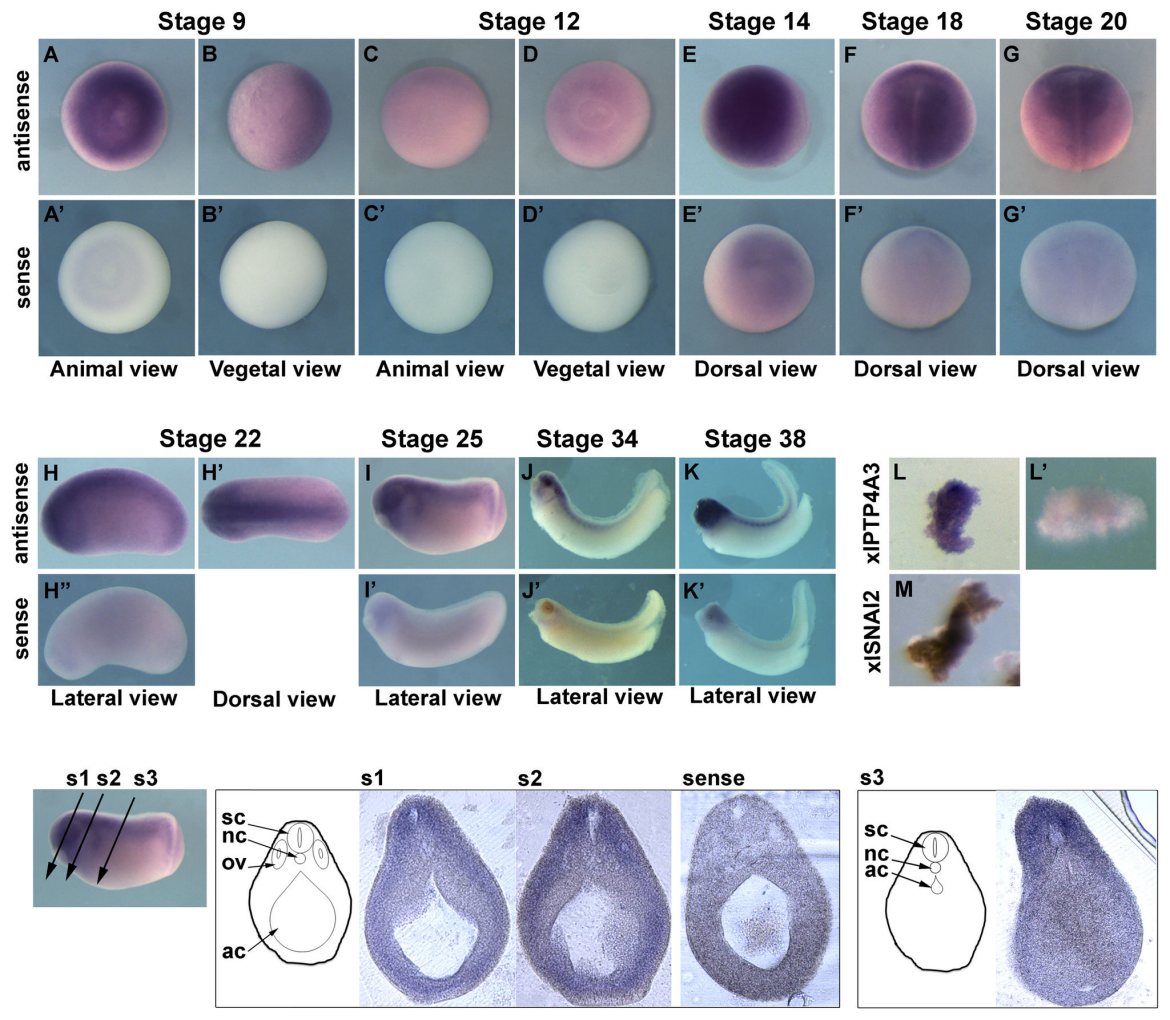
Spatial expression profile of xlPTP4A3 during embryogenesis. A-K) Whole-mount *in* *situ* hybridization showing the spatial expression of xlPTP4A3 during early *Xenopus* development. A’-K’) Corresponding negative control using a sense probe of xlPTP4A3 mRNA. L) Whole-mount *in* *situ* hybridization of xlPTP4A3 in NC explants dissected at stage 17. L’) Corresponding negative control using a sense probe of xlPTP4A3 mRNA. M) Whole-mount *in* *situ* hybridization of the NC marker xlSNAI2 on stage 17-dissected NC explants. s1-s3) Transverse sections through a stage 25 embryo stained by whole-mount *in* *situ* hybridization for xlPTP4A3 mRNA. Sense) Corresponding negative control using an xlPTP4A3 mRNA sense probe. sc: spinal cord, nc: notochord, ov: otic vesicle, ac: archenteron.

The PTP4A3 protein is conserved in *Xenopus laevis* and shares 89% identity with human PTP4A3 including the PTP catalytic site and the prenylation motif. PTP4A3 expression pattern was analyzed during early development of *Xenopus laevis* to investigate the role of PTP4A3 in embryos. We analyzed the global expression of xlPTP4A3 throughout embryogenesis using real-time PCR. xlPTP4A3 is expressed both maternally and zygotically throughout early developmental stages with a slight increase from stage 20 (data not shown). Whole-mount in situ hybridization (WISH) was performed at different embryonic stages to investigate the spatial expression pattern of xlPTP4A3. At late blastula stage (Stage 9, Figure 1, A-B), xlPTP4A3 was specifically detected in the animal hemisphere but not in the vegetal hemisphere of the embryo; no signal was observed with the sense control probe (Fig. 1, A’-B’). In late gastrulas (Stage 12, Figure 1, C-D), xlPTP4A3 expression decreased. As neurulation proceeded and NC cells were specified (stages 14-20), higher amounts of xlPTP4A3 transcripts were found along the dorsal midline, including the neural folds and the NC (Figure 1, E, F and G). From tailbud stage 22 to tadpole stage 38, xlPTP4A3 expression was observed along the dorsal midline and the craniofacial structures (Figure 1, Stage 22-38, H-H’, I, J and K). Very limited background was observed with the sense probe (Figure 1, E’-K’). Transverse sections through the head of stage 25 embryos showed xlPTP4A3 expression in the cephalic mesenchyme, which is of NC origin (Figure 1, s1-s2), and in trunk paraxial mesoderm cells (Figure 1, s3). No or little background was observed with the sense control probe (Figure 1, sense). WISH was conducted on stage 17-dissected NC explants to unambiguously demonstrate xlPTP4A3 expression in the NC. xlPTP4A3 (Figure 1, L), and the NC marker xlSNAI2 (xlSNAI2, Figure 1, M) were expressed in the NC explants but no signal was observed with the sense control probe (Figure 1, L’). These results demonstrate that xlPTP4A3 is expressed in NC territory during *Xenopus laevis* development. 

### Depletion of *xlPTP4A3* reduces NCC territory

**Figure 2:**
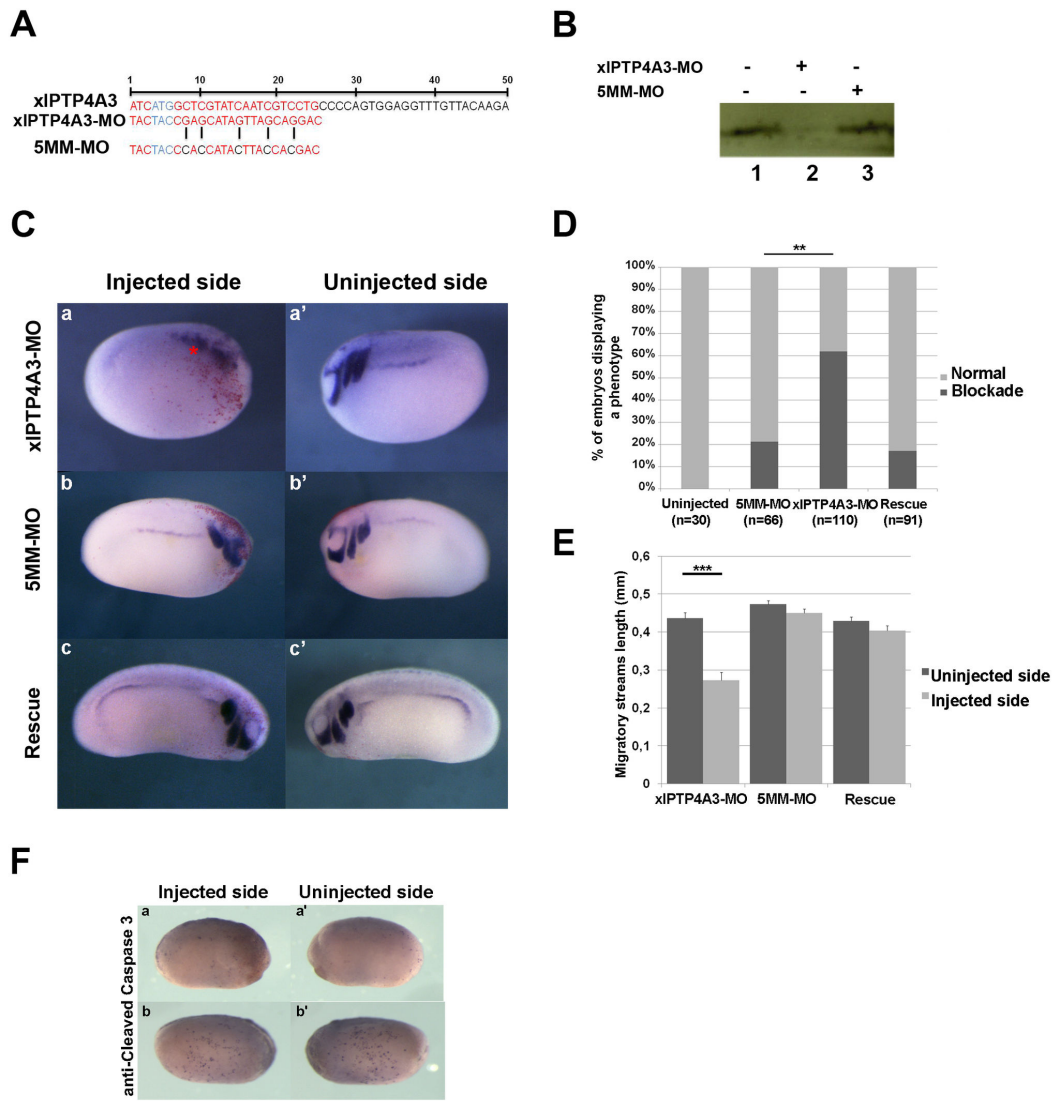
Depletion of xlPTP4A3 reduces NCC territory. Control 5MM-MO or xlPTP4A3-MO were injected at the four-cell stage in the animal pole region and embryos were cultured until stage 22. A) Morpholino sequence targeting xlPTP4A3. B) xlPTP4A3-MO specifically knocked down the translation of xlPTP4A3 *in* *vitro* using the TNT® Coupled Reticulocyte Lysate System. C) xlPTP4A3 depleted embryos showed blockade of NCC territory (a-a’) and can be rescued by co-injection of mouse mPTP4A3 (c-c’), as shown by whole-mount *in* *situ* hybridization on xlTWIST. The injected side was monitored using the co-injection of nuclear-targeted β-galactosidase (red dotted signal). Injection of the control 5MM-MO does not affect the NCCs territory, as in the control noninjected embryos (b-b’). D) Quantitative results of relative phenotype (**p<0,01). E) The phenotype quantification was determined as the length of the NC migratory streams showing that the migratory streams are shorter in the injected side than in the uninjected side (n=15) (***p<0,001). F) Immunodetection of cleaved caspase 3 on xlPTP4A3-MO injected embryos. Two phenotypes are observed: one of weaker apoptosis in the injected side compared to the uninjected side (a-a’) and one of no differential apoptosis between the two sides (b-b’).

We performed knock-down experiments using an antisense oligonucleotide morpholino directed against *X. laevis ptp4A3* (xlPTP4A3-MO) to investigate the physiological role of xlPTP4A3. The morpholino targeted the first 25 nucleotides of the xlPTP4A3 mRNA coding sequence, including the translation start codon, blocking translation (Figure 2A). A similar oligonucleotide morpholino with five mismatches (5MM-MO) was used to control the specificity of the phenotypes (Figure 2A). Since we did not have an antibody against *X. laevis* PTP4A3, we used the S-35 labeled Methionine TNT® Reticulocyte Lysate System, followed by SDS-PAGE, to test the efficiency of the two morpholinos *in vitro*. We designed a plasmid containing xlPTP4A3-MO target sequence in frame with EGFP sequence (xlPTP4A3-MO-EGFP). While 5MM-MO had no effect on the translation of xlPTP4A3-MO-EGFP mRNA (Figure 2B, lane 3), xlPTP4A3-MO efficiently prevented xlPTP4A3-MO-EGFP translation, (Figure 2B, lane 2). 

The injection of xlPTP4A3-MO into one blastomere at the four-cell stage blocked NCC migration on the injected side (as shown by WISH using *twist* as a marker for migratory NC in stage 22 embryos). No migratory streams could be observed in the injected side (Figure 2C, a) but clear streams were visible in the uninjected contralateral side (Figure 2C, a’). The extent of NC migration blockade was quantified as the percent of embryos where no migration was observed in the injected side (Figure 2D) and also as the length of the migratory streams in the injected side compared to the uninjected side (Figure 2E). The amount of injected xlPTP4A3-MO (40ng) was determined in a dose-response experiment. No difference in NCC migration was observed in the control 5MM-MO-injected embryos (Figure 2C, b-b’), suggesting that the observed effect is specifically due to PTP4A3 down-regulation. We examined whether mmPTP4A3 gain of function could rescue the normal phenotype to further confirm that the lack of PTP4A3 activity was responsible for the reduction in NCC migration. NCC migration defects were efficiently rescued by co-injection of wild type mmPTP4A3 with xlPTP4A3-MO (Figure 2C, c-c’). This was not observed upon co-injection of xlPTP4A3-MO with the mmPTP4A3(C104S) mutant which is devoid of pro-migrating activity [5] (data not shown). Reduced NC territory in the injected side could be due to an apoptotic process induced by the reduction of xlPTP4A3 level, as shown in lung cancer cells [24]. Immunodetection of activated-caspase 3 in injected embryos with xlPTP4A3-directed morpholino was carried out to test this hypothesis. Weak apoptosis was observed at stage 22 similarly in the injected area and control embryos; the level of apoptosis observed is probably not sufficient for this to be the main cause of the reduction in NC area (n=16) (Figure 2F, a-a’ and b-b’). Furthermore, immunodetection of activated-caspase 3 on sections of the same embryos was performed to assess apoptosis specifically in the NC domain. No significant differences on apoptosis between the injected side and the uninjected side are observed (data not shown). 

### PTP4A3 gain of function enlarges the NC domain *in vivo*

**Figure 3:**
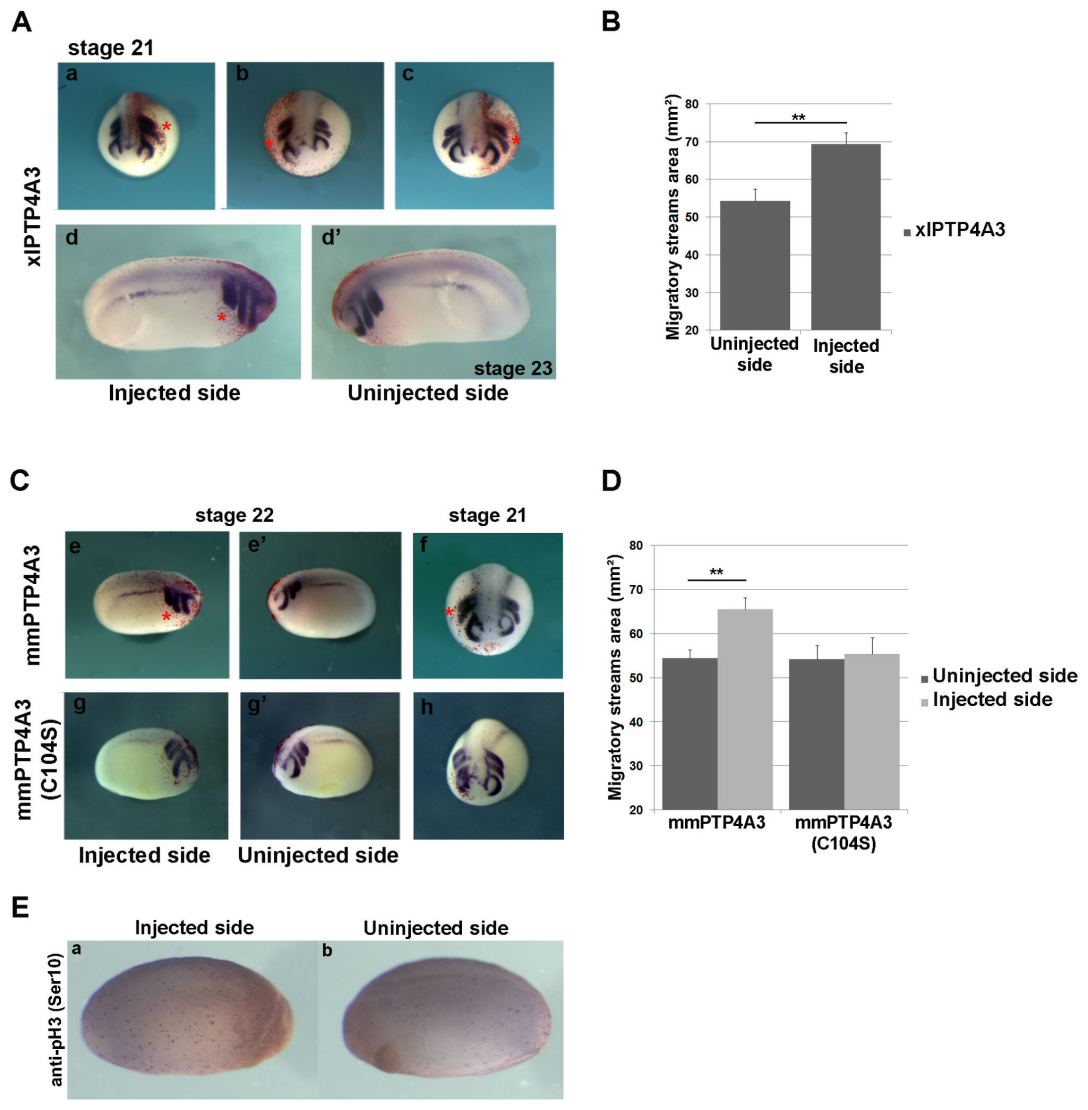
xlPTP4A3 gain of function induces strengthening of the anterior NC migration streams *in* *vivo*. xlPTP4A3, mmPTP4A3 or the mutant form mmPTP4A3(C104S) were injected at the four-cell stage in the animal pole region, and embryos were cultured until stage 22. A) Overexpression of xlPTP4A3 induces a strengthening (b, d *vs*. d’) and/or lengthening (a, c) of the anterior NC migration streams as showed by whole-mount *in* *situ* hybridization on xlTWIST. B) xlPTP4A3 corresponding phenotype quantification was determined as the area of the NC migratory streams showing that the migratory streams surface is greater in the injected side than in the uninjected side (n=15) (**p<0,01). C) Overexpression of mmPTP4A3 causes a strengthening and/or lengthening (e *vs*.e’, f) of the anterior NC migration streams while overexpression the mutant form mmPTP4A3(C104S) does not seem to significantly affect the migration of the NC (g *vs*.g’, h). D) mmPTP4A3 or mmPTP4A3(C104S) corresponding phenotype quantification was determined as the area of the NC migratory streams showing that mmPTP4A3 injected embryos display a greater anterior NC area in the injected side than in the uninjected side while no difference in the anterior NC area could be observed in the mmPTP4A3(C104S) injected embryos between the two sides (n=15) (**p<0,01). E) Immunodetection of phosphoHistone 3 (Ser10) on xlPTP4A3 gain of function injected embryos. Injection of xlPTP4A3 does not seem to affect cell proliferation in the injected side relative to that of the uninjected side (a *vs*.b).

PTP4A3 down-regulation reduced NCC migration and so we tested whether increased NCC migration could be stimulated by gain of function experiments. We tested the role of PTP4A3 function in anterior NCC migration using a HA-tagged xlPTP4A3 gain of function experiment. Injection of HA-xlPTP4A3 in one blastomere at two-cell stage embryos induced an enlarged NC domain (Figure 3A, a-d), as shown by xlTWIST expression at stage 21-23 (45% of embryos displayed this phenotype on the injected side, n=46). The extent of NC strengthening was quantified as the area of the migratory streams in the injected side compared to the uninjected side (Figure 3B). Similar results were obtained upon injection of mmPTP4A3 mRNA, but not with injection of the mutant mmPTP4A3(C104S) (Figure 3C, e-e’ and f vs. g-g’ and h, n=66 and n=38 respectively). In the same way, the extent of NC strengthening was quantified as the area of the migratory streams in the injected side compared to the uninjected side (Figure 3D). We tested whether xlPTP4A3 overexpression increased cell proliferation, using phosphoHistone 3 (Ser10) immunodetection (PTP4A3 expressing UM did not show an increase in cell proliferation when compared to the inactive PTP4A3(C104S) mutant, but proliferative response seems to depend on the cell type [25] [24]). We found no difference between the injected and uninjected side of stage 22 embryos in gain of function experiment (n=22) (Figure 3E, a-b). Furthermore, immunodetection of phosphoHistone 3 (Ser10) on sections of the same embryos was performed to assess proliferation specifically in the NC domain. No significant differences on proliferation between the injected side and the uninjected side are observed (data not shown). These results, taken together, indicate that PTP4A3 regulates NCC migration *in vivo*. 

### PTP4A3 effect on NCC migration is cell-autonomous and depends on its phosphatase activity

**Figure 4:**
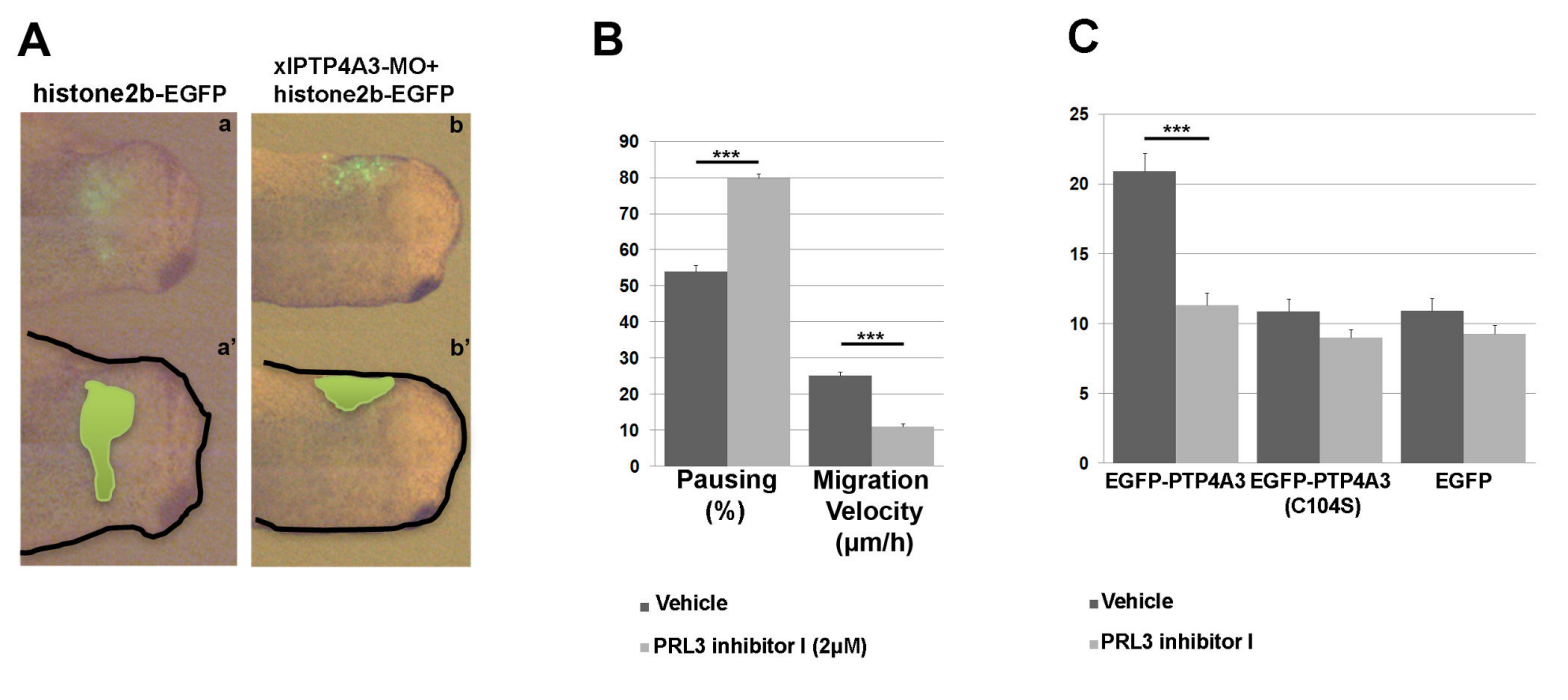
Depletion of xlPTP4A3 inhibits both migration of NCC and uveal melanoma cells. A) Isotopic and isochronic grafts of xlPTP4A3-depleted NC explants into host embryos showed a delayed NCC migration (b-b’) when compared to the control histone 2b-GFP NC explants (a-a’). B) Timelapse videomicroscopy of stage 17-derived NCC treated with a pharmacological inhibitor of PTP4A3, a rhodanine derivative (PRL-3 inhibitor I), shows that inhibition of PTP4A3 increases the average of pausing NC cells and strongly decreases the migration velocity of the treated cells (relative to that of vehicle treated cells). The result is representative of three independent experiments and the average of counted cells is 60 cells per condition (***p<0,001). C) Timelapse videomicroscopy of uveal melanoma OCM1 cells stably expressing EGFP-PTP4A3, EGFP-PTP4A3(C104S) or EGFP on collagen I shows that inhibition of PTP4A3, using PRL-3 Inhibitor I, decreases specifically the migration velocity of the PTP4A3 expressing cells (relative to the velocity of untreated and control cells). The result is representative of three independent experiments and the average of counted cells is 50 cells per condition (***p<0,001).

Isochronic and isotopic NC grafting experiments were performed to analyze if the defect in xlPTP4A3 morphants was cell-autonomous in the NC or due to a defective migration environment. Whole-embryo depletion of PTP4A3 (by co-injections of xlPTP4A3-MO and histone2b-GFP, n=9) was carried out as previously described and the NC was isolated at stage 17. Morphant NC was then back-grafted into stage 17 sibling control host embryos. Control NC was injected with histone2b-GFP alone (n=10). At stage 22 (about 18h after grafting), the morphant NCC had remained at the grafted site (Figure 4A, b-b’) but control grafts exhibited efficient migration (Figure 4A, a-a’). 

We then tested if the role of PTP4A3 in NC migration was dependent on its phosphatase activity using a pharmacological inhibitor, PRL-3 Inhibitor I (Sigma #P0108). PRL-3 Inhibitor I specifically reduced uveal melanoma cell OCM1 EGFP-PTP4A3 migration on collagen I, to the value observed for the mutant OCM1 EGFP-PTP4A3(C104S) or OCM1 EGFP expressing cells (Figure 4C). We conducted time-lapse videomicroscopy on NCC explants cultured on fibronectin *in vitro*, in the presence or absence of this inhibitor, which showed that the phosphatase activity of PTP4A3 influenced NCC migration. We observed that individual cells detached from the control NC explants (cultured in vehicle only), produced protrusions and actively migrated. Treated NCC showed a significantly lower migration velocity, and more pausing than the untreated NCC (Figure 4B). PTP4A3 biochemical function is therefore essential for NCC migration, both *in vivo* and *in vitro*. 

## Discussion

Overexpression of the dual phosphatase PTP4A3 in UM is associated with reduced patient survival [5]. Expression of PTP4A3 in UM cell lines increased migration and invasive properties of these cells [5]. PTP4A3 has been associated with the EMT process that occurs in tumors [26] and SNAI1, a key transcription factor involved in EMT, is a positive regulator of PTP4A3 [27]. UM derives from the transformation of anterior NC, suggesting that PTP4A3 could be a protein involved in NC EMT *in vivo* (like snail1/2 or twist). We have demonstrated that xlPTP4A3 transcripts are expressed in the NC territory at the time of NC formation *in vivo*, in *Xenopus laevis* embryos. Due to the lack of antibodies against *X. laevis* PTP4A3, we performed immunodetection of the protein in migrating quail NCC *in vitro* which showed that PTP4A3 is indeed present in NCC (data not shown). Knock-down after xlPTP4A3-directed morpholino injection reduced xlTWIST expression (another transcription factor involved in EMT, [28]). This effect could be rescued by mmPTP4A3, but not by the phosphatase-dead PTP4A3(C104S) mutant which is devoid of pro-migratory potential. We explored whether the lack of xlPTP4A3 is responsible for the NC domain reduction by gain of function experiment to see if it would produce the opposite effect. We observed a larger xlTWIST expressing NC area in the injected side. The observed increase in NC domain could be due to increased recruitment of naïve cells toward a NC phenotype, or increased cell migration. We show that the migration of the cells is clearly affected *in vivo*. This was shown by isochronic and isoallelic graft experiments, and *in vitro* NC explants cultured in the presence of PTP4A3 chemical inhibitors (all the aminoacids described to interact with the drug [29] are conserved in the xlPTP4A3). PTP4A3 does not seem to be essential for NC development in mammals since its targeted depletion in mice does not compromise development. Similarly, fetal heart tissue expresses high levels of PTP4A3 and can develop normally in absence of PTP4A3 [30]. Lin et al. suggest that, in zebrafish, most of the PTP4A1 and PTP4A2 are expressed in the neuronal cell lineage and that PTP4A3 has evolved a more specific expression in the mesodermal cell lineage [7]. Our results suggest that in *Xenopus laevis* PTP4A3 is still expressed in cells of ectoderm origin (the NC cells) which may be considered as a transitory expression state between the central nervous system and mesodermal lineage. We propose that PTP4A3 participates in the emigration of the anterior NCC *in vivo* through its role in the control of cell migration. Thus, understanding PTP4A3 mechanism of action during NC migration may provide insight into PTP4A3 related migratory and invasive phenotypes in human uveal melanoma pathology. In that way, the tractability of *Xenopus laevis* embryos could be used to test the biological relevance of mammalian PTP4A3 targets. 
